# Localization-delocalization wavepacket transition in Pythagorean aperiodic potentials

**DOI:** 10.1038/srep32546

**Published:** 2016-09-02

**Authors:** Changming Huang, Fangwei Ye, Xianfeng Chen, Yaroslav V. Kartashov, Vladimir V. Konotop, Lluis Torner

**Affiliations:** 1 Department of Physics and Astronomy, Shanghai Jiao Tong University, Shanghai 200240, China; 2 Key Laboratory for Laser Plasma (Ministry of Education), IFSA Collaborative Innovation Center, Shanghai Jiao Tong University, Shanghai 200240, China; 3 ICFO-Institut de Ciencies Fotoniques, The Barcelona Institute of Science and Technology, 08860 Castelldefels (Barcelona), Spain; 4 Institute of Spectroscopy, Russian Academy of Sciences, Troitsk, Moscow Region, 142190, Russia; 5 Centro de Física Teórica e Computacional and Departamento de Física, Faculdade de Ciências, Universidade de Lisboa, Campo Grande 2, Edifício C8, Lisboa 1749-016, Portugal; 6 Universitat Politecnica de Catalunya, 08034, Barcelona, Spain

## Abstract

We introduce a composite optical lattice created by two mutually rotated square patterns and allowing observation of continuous transformation between incommensurate and completely periodic structures upon variation of the rotation angle *θ*. Such lattices acquire periodicity only for rotation angles cos *θ* = *a*/*c*, sin *θ* = *b*/*c*, set by Pythagorean triples of natural numbers (*a*, *b*, *c*). While linear eigenmodes supported by lattices associated with Pythagorean triples are always extended, composite patterns generated for intermediate rotation angles allow observation of the localization-delocalization transition of eigenmodes upon modification of the relative strength of two sublattices forming the composite pattern. Sharp delocalization of supported modes for certain *θ* values can be used for visualization of Pythagorean triples. The effects predicted here are general and also take place in composite structures generated by two rotated hexagonal lattices.

The formation and evolution of localized excitations in inhomogeneous systems, governed by the Schrödinger equation^1^, is of paramount importance for understanding a variety of fundamental physical phenomena. These include quantum particles in the external potentials, matter waves in traps^2^,^3^,^4^, evolution of optical pulses^5^ and beams^6^. The dynamics of such systems are determined by the properties of the corresponding potentials. Thus, periodic potentials support only delocalized Bloch waves in the allowed bands of the spectrum^7^, while in disordered potentials all eigenmodes can be localized^8^. Yet another scenario is encountered in aperiodic potentials which, however, feature long-range order, such as fractals^9^ or quasi-crystals^10^,^11^,^12^. Eigenmodes in one-dimensional (1D) aperiodic systems may exhibit localization-delocalization transition (LDT) upon smooth deformation of the underlying potential^13^,^14^,^15^,^16^,^17^,^18^,^19^, a behavior that places them between periodic and fully disordered systems.

The fundamental relevance of aperiodic structures featuring long-range order became obvious after discovery of quasi-crystals^10^ in experiments on electron diffraction^20^. Nowadays these and other types of aperiodic structures are widely studied in different areas of science^21^,^22^. Especially rich opportunities for experimentation with aperiodic structures appear in optics^23^ and matter-waves^24^, where quasi-crystal-like potentials can be induced by several interfering plane waves in reconfigurable geometries or fabricated in suitable materials^25^,^26^.

The phenomenon of LDT was predicted upon analysis of the tight-binding model of incommensurable potentials^13^ and in the framework of the Harper (alias Aubry-Andre) model^14^,^15^,^16^,^17^,^18^,^19^, for which the existence of LDT was supplied by a mathematical proof 27. In particular, wave localization in 1D quasi-crystals mediated by variation of their parameters was observed experimentally in optics^28^,^29^ and in Bose-Einstein condensates^24^. LDT may also take place in dissipative incommensurable 1D lattices, obeying parity-time symmetry^30^. The existence of LDT in certain 1D aperiodic potentials, however, does not guarantee that the effect occurs in higher dimensions. Some experiments show diffraction in 2D aperiodic structures^11^,^12^,^31^,^32^, while others^23^,^33^,^34^,^35^,^36^ indicate the formation of localized modes.

In this paper we introduce aperiodic potentials built as a superposition pattern of two identical periodic sublattices (either square or hexagonal) mutually-rotated by an angle *θ*. By changing *θ* the potential can be continuously transformed from periodic to aperiodic geometries and *vice versa*, without any change to its rotational point symmetry. The restoration of periodicity may occur at an infinite set of the rotation angles given by Pythagorean triples, at which the linear modes turn into Bloch waves. For hexagonal sublattices, we uncover new triples leading to periodicity restoration. Using such potentials we establish the existence of previously elusive 2D LDT. Furthermore, in our case LDT occurs not only upon variation of the relative depth of two sublattices, but also upon variation of the rotation angle. We show how the restoration of periodicity affects the thresholds for formation of self-sustained solitary waves previously studied only in quasi-crystals with predetermined symmetry^23^,^37^,^38^,^39^,^40^,^41^,^42^,^43^,^44^. The results obtained are directly applicable both in optics, where an aperiodic refractive index can be induced in various materials^45^,^46^,^47^,^48^,^49^, and in Bose-Einstein condensates which can be manipulated by optical lattices^2^,^3^,^4^. In view of the recent interest in moiré patterns resulting from two superimposed honeycomb lattices with slightly different parameters, like graphene on hexagonal Boron Nitride(hBN)^50^,^51^,^52^,^53^, we emphasize that the aperiodic structures reported are based on identical sublattices allowing restoration of periodicity upon change of the rotation angle.

Incommensurability is at the heart of construction of aperiodic potentials. It is also one of the most important objects in number theory, which has been in use since the time of the ancient Greeks^54^. The celebrated Pythagorean theorem is intimately related to incommensurability: it gives rise to so-called Pythagorean triples, i.e. natural numbers (*a*, *b*, *c*) satisfying the condition *a*^2^ + *b*^2^ = *c*^2^ and setting lengths of catheti and hypotenuses of a right (Pythagorean) triangle. There are 16 primitive Pythagorean triples with *c* < 100, including (3, 4, 5), (5, 12, 13), (8, 15, 17), etc. These are directly connected with the transition between the fully periodic and aperiodic geometries introduced here. For example, consider a potential *V*(**r**) [hereafter **r** = (*η*, *ζ*) is a two-dimensional (2D) transverse position vector with *η* and *ζ* being spatial coordinates] created by two basic square lattices with equal periods that are mutually rotated by an angle of *θ*:


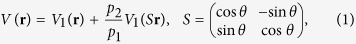


where *V*_1_(**r**) = *p*_1_[cos(2*η*) + cos(2*ζ*)] is one of the sub-lattices, and *p*_1_ and *p*_2_ are the sublattice depths. The potential (1) is aperiodic for all values of *θ* except when Pythagorean triangles are formed, i.e. when cos *θ* = *a*/*c* and sin *θ* = *b*/*c*, where (*a*, *b*, *c*) is a Pythagorean triple. In this last case the periodicity of potential is restored^55^. Examples of such lattices – called Pythagorean lattices – are shown in Fig. 1. Each Pythagorean lattice possesses a square primitive cell (see ref. 55 for technical details), whose area depends on the Pythagorean triple defining it. For all other rotation angles the potential *V*(**r**) exhibits an aperiodic structure with long-range order (shown in the central panel of Fig. 1). Therefore, variation of *θ* causes a smooth transformation between periodic and quasi-periodic structures, while the underlying four-fold rotation symmetry is *always preserved*. Modifications in the lattice depths *p*_1_ and *p*_2_ do not affect the four-fold lattice symmetry either, thereby making the potential (1) particularly convenient to study the occurrence of LDT phenomena.

We note that one standard approach to create optical quasi-crystals relies on the superposition of *N* plane waves, with *N* being odd and selected such that the pattern in principle cannot be periodic. In our case we use two sets of four plane waves that are mutually-rotated. Their superposition can give rise to a periodic distribution for certain rotation angles and to aperiodic distributions for all other angles (the possibility of generating quasi-crystals in the sense of the generally accepted definition^11^,^12^ remains an open question).

Let us now consider a Pythagorean lattice as an optical potential for light propagation. In a paraxial approximation, a light beam with amplitude *q* in a medium with the shallow refractive index modulation (1) is governed by the dimensionless Schrödinger equation


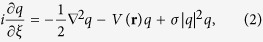


which accounts for focusing (defocusing) Kerr nonlinearity at *σ* = −1 (*σ* = +1), and becomes linear at *σ* = 0. Here ∇ ≡ (∂/∂*η*, ∂/∂*ζ*) and *ξ* is the propagation distance.

The eigenmodes of the linear system (i.e. when *σ* = 0) are searched for in the form *q*(*η*, *ζ*, *ξ*) = *w*(*η*, *ζ*)*e*^*iβξ*^, where the function *w*(*η*, *ζ*) describes the mode profile and *β* is its propagation constant. The degree of transverse localization of the modal field can be characterized by the integral form-factor 
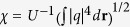
, where *U* = ∫|*q*|^2^*d***r** is the energy flow. The form-factor is inversely proportional to the mode width. Hence, higher *χ* means stronger localization.

One of our main results is illustrated in Figs 2 and 3. It consists of observation of the LDT in a 2D aperiodic structure created by two rotated square lattices. Figure 2 shows representative dependencies of the form-factor of the eigenmode with largest *β* on the rotation angle *θ* (here *θ* ∈ [0, *π*/2]) and on the depth *p*_2_ of the second lattice.

For a fixed *p*_1_ a relatively sharp LDT occurs when *p*_2_ exceeds certain critical value [see typical dependence *χ*(*p*_2_) in Fig. 3(a) and associated transformation of mode profiles in Fig. 4(a) for the angle tan *θ* = 3^−1/2^ corresponding to an aperiodic potential]. Since gradual transition from delocalized to strongly localized modes occurs within a narrow interval of *p*_2_ values, we define the LDT threshold as a *p*_2_ value at which form-factor *χ* exceeds 0.1. The LDT threshold depends on depths of both sublattices *p*_1_, *p*_2_ and not on their ratio, as is obvious from Fig. 3(b). The lesser the depth of one sub-lattice the deeper the other sub-lattice should be for the onset of localization. Surprisingly, however, the threshold depends very weakly on the rotation angle [see weakly varying boundary between blue and green/red domains in Fig. 2(a,b)].

When the rotation angle coincides with a Pythagorean angle the potential periodicity is restored and all modes become delocalized regardless of *p*_2_ as they represent conventional Bloch states [see lower curve in Fig. 3(a)]. Such Pythagorean angles are clearly identified in Fig. 2 by the location of narrow vertical (blue) delocalization stripes. Thus, Pythagorean triples *can actually be visualized* by capturing the linear diffraction patterns produced by narrow inputs: even if *p*_2_ is above the LDT threshold, a sudden delocalization of the output pattern occurs for rotation angles *θ* coinciding with any Pythagorean angle. This is shown in Fig. 4(b,c), which compare linear propagation of narrow Gaussian beams in aperiodic (tan *θ* = 3^−1/2^) and Pythagorean (tan *θ* = 3/4) potentials. Note the four-fold rotation symmetry exhibited by the linear diffraction pattern in Fig. 4(c).

In Fig. 5(a) we show the band-gap spectrum of the Pythagorean lattice corresponding to tan *θ* = 3/4 [the triple (3, 4, 5)]. Note that the *α* = 1 band is remarkably flat. Since the effective diffraction strength is determined by the band curvature, the flatness causes an anomalously slow broadening of the beam that excites modes from this flat band. Such effect, which was observed earlier in other lattice types^56^,^57^,^58^, is also well observable in the Pythagorean lattice as seen in Fig. 4(c). Note that the input standard Gaussian beams that we use here excite mostly modes from the top flat band, while they do not excite modes from the lower bands that are not necessarily flat.

Returning to new possibilities afforded by the smooth one-parametric transition between periodic and aperiodic geometries, one may wonder what happens to the linear spectrum when the transition takes place. The answer is given in Fig. 5(b), which shows the evolution of 600 largest eigenvalues *β*_*k*_ of the system (corresponding modes are calculated on the [−80*π*, +80*π*] window with zero boundary conditions) when the deviation of the rotation angle from a Pythagorean angle increases. The gap between the first and second groups of eigenvalues (former *α* = 1 and *α* = 2 bands) does not disappear abruptly and it only closes completely for deviations in *θ* of the order of 0.5 degrees. Therefore, phenomena associated with the presence of forbidden gaps can occur even in slightly aperiodic lattices. Thus, in a nonlinear system the persistence of a finite gap for the slight detuning of the rotation angle from a Pythagorean implies that the energy flow threshold for gap soliton existence does not disappear abruptly upon detuning.

The potential (1) also has an impact on the properties of nonlinear localized states. It has been proven^59^ that in the focusing 2D nonlinear Schrödinger equation with a periodic potential, a minimal energy flow *U* is required to create a 2D soliton. This is applicable to model (2) with a Pythagorean lattice *V*(**r**) and with *σ* = −1. On the other hand, if a system supports localized linear modes, one can expect that solitons may bifurcate from such modes with an increase of the peak amplitude. Thus, for periodic and aperiodic potentials one expects qualitatively different behavior of the *U*(*β*) curves (here *β* is the propagation constant of soliton *q* = *w*(*η*, *ζ*)*e*^*iβξ*^). This is confirmed by Fig. 6(a) for *σ* = −1. Indeed, for tan *θ* = 3/4 corresponding to periodic potential (black curves 1 and 2), a minimal energy flow is required for soliton formation irrespectively of *p*_2_ value. However, for tan *θ* = 3^−1/2^ the *U*(*β*) curves are qualitatively different below (red curve 1) and above (red curve 2) LDT threshold in *p*_2_: in the former case minimal energy flow is still needed to form a soliton, while in the latter case energy flow goes to zero indicating a bifurcation from linear mode. Solitons are found to be stable for the intervals where *dU*/*dβ* > 0^55^, similarly to prediction of the Vakhitov-Kolokolov stability criterion. In a Pythagorean lattice with defocusing nonlinearity (*σ* = +1) solitons may form in finite gaps, even for a small detuning of the rotation angle from a Pythagorean one [see Fig. 6(d) for the corresponding *U*(*β*) curve]. Such solitons feature an energy flow threshold and are stable in the largest part of the gap, except for narrow regions close to the gap edges. Note the unusual symmetry of the soliton shapes supported by the composite lattices [Fig. 6(b,c)].

The above results are general in the sense that the mutual rotation of two geometrically identical structures (of any symmetry) sets the basis for the construction of one-parametric 2D potentials allowing continuous transition between periodic and aperiodic geometries and, hence, observation of LDT. To illustrate the generality of the effect we consider the potential (1) composed of two rotated hexagonal (triangular) lattices 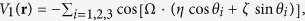 where *θ*_*i*_ = 0, 2*π*/3, 4*π*/3. Examples of such composite potentials are given in Fig. 7. By analogy with Pythagorean triples, it is possible to introduce a triple of positive integers (*a*, *b*, *c*), such that *c*^2^ = *a*^2^ + *b*^2^ + *ab*, which uniquely defines the rotation angle (its tangent is given by 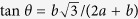) at which periodicity is restored. Such triples are different from Pythagorean triples^55^. The corresponding lattices also feature LDT, while restoration of lattice periodicity for suitable rotation angles leads to delocalization depths in the *π*/3-periodic *χ*(*θ*) dependencies qualitatively similar to those encountered in square rotated lattices^55^.

## Conclusions

In summary, we have shown that LDT can occur in a new class of *two-dimensional* composite lattices created by the superposition of two mutually-rotated periodic structures. Even above the LDT threshold for given amplitudes of the sub-lattices, where all eigenmodes are localized for the majority of rotation angles, one observes *sharp delocalization* for rotation angles corresponding to Pythagorean triples. Thus, for specific rotation angles *θ* allowing periodicity restoration one always gets delocalization, while for *θ* values leading to aperiodic lattices localization occurs for *p*_1_, *p*_2_ values taken above the LDT threshold. Since this conclusion is based on general arguments we anticipate that the localization-delocalization transition can be observed in structures with different internal symmetries and can be experimentally realized in various systems, including optical settings and Bose-Einstein condensates. The nature of the underlying composite linear lattices also has an impact on the properties and symmetries of nonlinear self-sustained excitations, allowing, for example, thresholdless creation of *two-dimensional* solitons in media with Kerr nonlinearity.

## Additional Information

**How to cite this article**: Huang, C. *et al.* Localization-delocalization wavepacket transition in Pythagorean aperiodic potentials. *Sci. Rep.* **6**, 32546; doi: 10.1038/srep32546 (2016).

## Supplementary Material

Supplementary Information

## Figures and Tables

**Figure 1:**
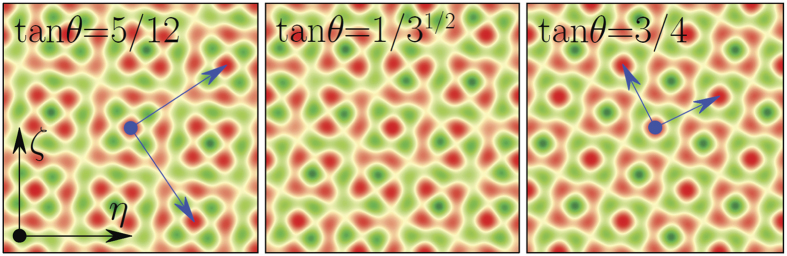
Lattices obtained by mutual rotation of two square lattices by an angle *θ* corresponding to the (5, 12, 13) and (3, 4, 5) triples (left and right panels, respectively) and of an aperiodic potential (central panel). The blue arrows show the primitive vectors. Lattices described by (1) are shown within the *η*, *ζ* ∈ [−4*π*, 4*π*] window for *p*_1_ = 1 and *p*_2_ = 0.5.

**Figure 2:**
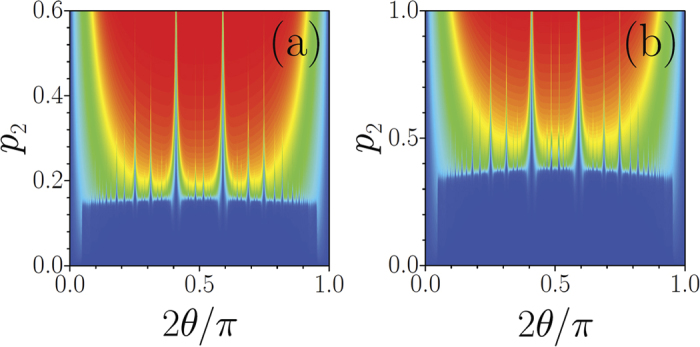
LDT illustrated by *χ*(*θ*, *p*_2_) dependencies for (**a**) *p*_1_ = 1 and (**b**) *p*_1_ = 0.5. Blue domains correspond to completely delocalized states and green/red domains correspond to localized modes.

**Figure 3:**
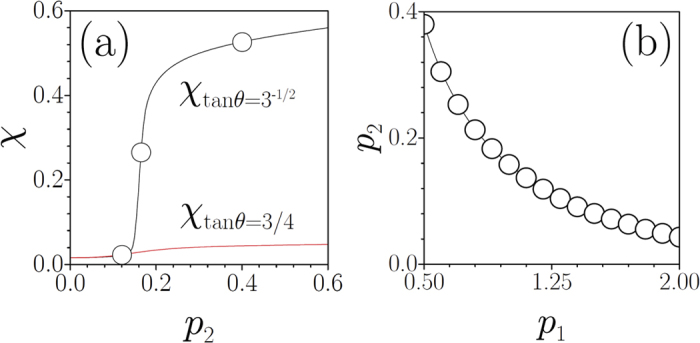
(**a**) Form-factor *vs p*_2_ for periodic (red curve) and quasi-periodic (black curve) lattices at *p*_1_ = 1. Circles correspond to linear modes from Fig. 4(a). (**b**) LDT threshold on (*p*_1_, *p*_2_)-plane defined as a line where *χ* = 0.1 at *θ* = *π*/6.

**Figure 4:**
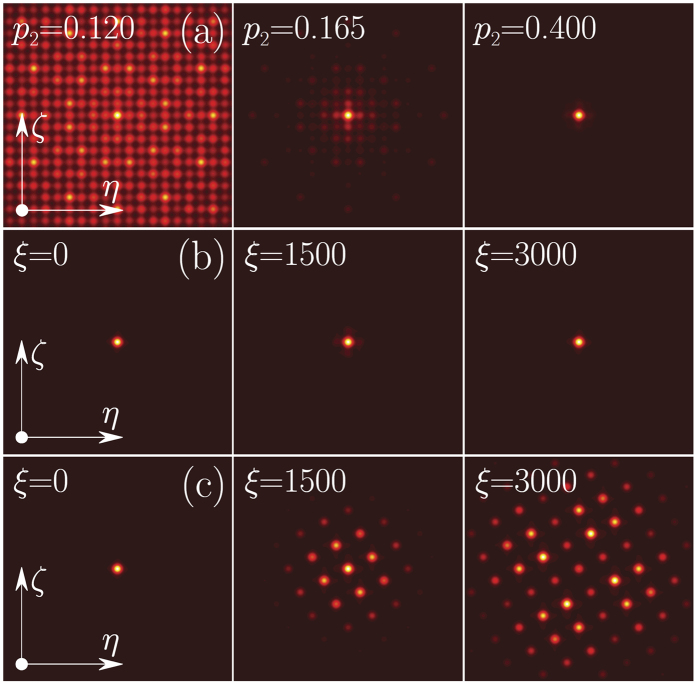
(**a**) Field modulus distributions in the linear mode with the largest propagation constant supported by the lattice with tan*θ* = 3^−1/2^ and *p*_1_ = 1 for different *p*_2_. Field modulus distributions for a single-site input beam at three different distances in the aperiodic potential with tan*θ* = 3^−1/2^ (**b**) and in the Pythagorean lattice with tan*θ* = 3/4 (**c**) at *p*_1_ = 1 and *p*_2_ = 0.4.

**Figure 5:**
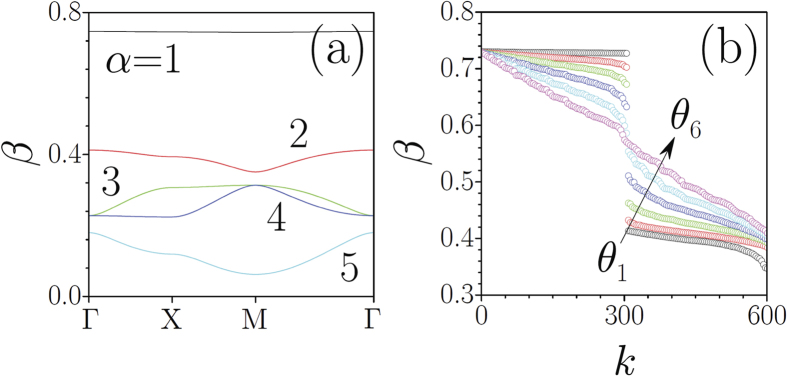
(**a**) Band-gap spectrum of a periodic lattice with tan*θ* = 3/4. (**b**) The transformation of discrete spectrum *β*_*k*_ of aperiodic lattice upon increase of the rotation angle from *θ*_1_ = arctan(3/4) + *π*/1800 (black circles) to *θ*_6_ = arctan(3/4) + 6*π*/1800 (magenta circles) in equal steps of *π*/1800. In both cases *p*_1_ = 1, *p*_2_ = 0.4.

**Figure 6:**
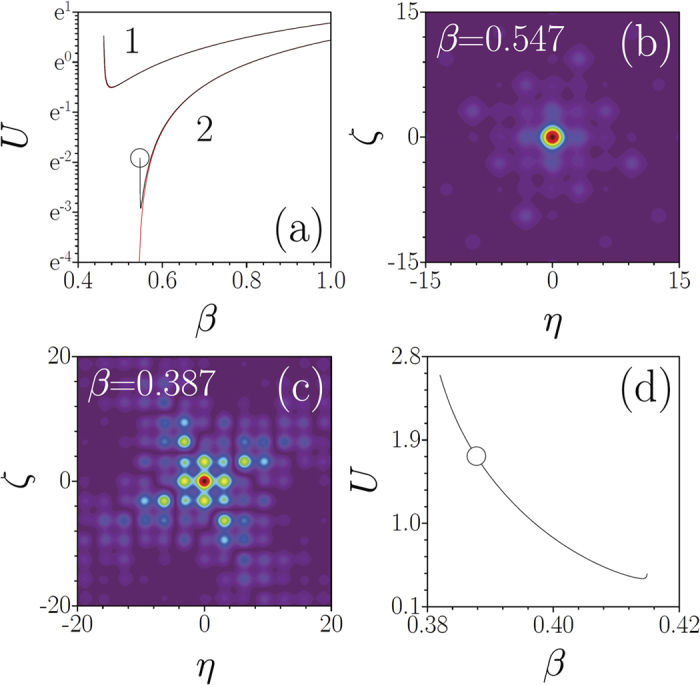
Energy flow *U vs β* for solitons in focusing media at *p*_2_ = 0.05 (curve 1) and *p*_2_ = 0.22 (curve 2). The black and red curves correspond to lattices with tan*θ* = 3/4 and tan*θ* = 3^−1/2^. (**b**) Soliton corresponding to the circle in (**a**). (**c**) Gap solitons in defocusing media corresponding to the circle in (**d**). (**d**) *U*(*β*) dependence for gap solitons in the lattice with *p*_2_ = 0.1 and *θ* = arctan(3/4) + *π*/900. In all cases *p*_1_ = 1. Panels (**b,c**) show field modulus distribution.

**Figure 7:**
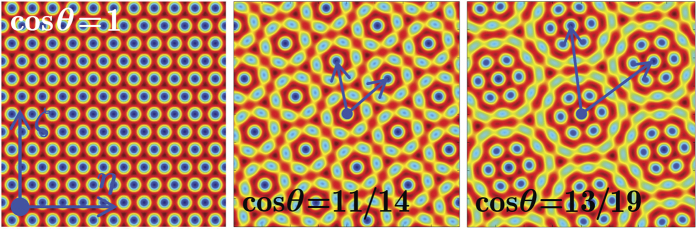
Potentials obtained mutual rotations of two hexagonal lattices for cos *θ* = 1 (left), cos *θ* = 11/14 (center) and cos *θ* = 13/19 (right). The lattices are shown within the *η*, *ζ* ∈ [−20, 20] window for *p*_1_ = *p*_2_ = 1. The blue arrows in the central and right panels are the primitive vectors.
