# Supplementary material for: Localization-delocalization wavepacket transition in Pythagorean aperiodic potentials
Source: Sci Rep. 2016 Sep 2;6:32546. doi: 10.1038/srep32546 (PMC5009312; doi:10.1038/srep32546)
Supplement: Supplementary Information [file srep32546-s1.pdf]

Supplementary Material for
Localization-delocalization wavepacket transition in Pythagorean aperiodic potentials

Changming Huang[1,2], Fangwei Ye[1,2], Xianfeng Chen[1,2], Yaroslav V. Kartashov[3,4], Vladimir V. Konotop[5], Lluis Torner[3,6]

[1] *Department of Physics and Astronomy, Shanghai Jiao Tong University, Shanghai 200240, China*
[2] *Key Laboratory for Laser Plasma (Ministry of Education), IFSA Collaborative Innovation Center, Shanghai Jiao Tong University, Shanghai 200240, China*
[3] *ICFO-Institut de Ciencies Fotoniques, The Barcelona Institute of Science and Technology, 08860 Castelldefels (Barcelona), Spain*
[4] *Institute of Spectroscopy, Russian Academy of Sciences, Troitsk, Moscow Region, 142190, Russia*
[5] *Centro de Física Teï¿œrica e Computacional and Departamento de Física, Faculdade de Ciï¿œencias, Universidade de Lisboa, Campo Grande 2, Edifício C8, Lisboa 1749-016, Portugal*
[6] *Universitat Politecnica de Catalunya, 08034, Barcelona, Spain*

(Dated: July 25, 2016)

In this Supplementary Material we summarize some basic properties of Pythagorean potentials and potentials composed of hexagonal (triangular) sub-lattices. With each of these potentials it is possible to connect a discrete system (lattice) having the same symmetry. Thus, to avoid terminological confusion in this Supplementary Material, we distinguish between the terms "potential" and "lattice", which refer to continuous and discrete structures, respectively. The lattices are presented as "atoms" located in the points where corresponding potential acquires local maxima, with straight connections between nearest neighbors that stress the symmetry of each sub-lattice and of the resulting composite structure. A more rigorous definition is given below. In the last part of the Supplementary Material we present the examples of soliton propagation in Pythagorean and aperiodic potentials.

I. PYTHAGOREAN POTENTIAL IS PERIODIC

Let us consider a 2D periodic potential $V_1(\mathbf{r})$, whose spatial periods are defined by the unit vectors $\mathbf{i}$ and $\mathbf{j}$, which are mutually orthogonal. In other words we require that

$$V_1(\mathbf{r}) = V_1(\mathbf{r}+\mathbf{i}) = V_1(\mathbf{r}+\mathbf{j}), \quad \text{and} \quad \mathbf{i}\cdot\mathbf{j} = 0. \tag{1}$$

We also define a "rotated" potential

$$V_2(\mathbf{r}) = pV_1(S\mathbf{r}), \quad \text{where} \quad S = \begin{pmatrix} \cos\theta & -\sin\theta \\ \sin\theta & \cos\theta \end{pmatrix} \tag{2}$$

where θ is the rotation angle and p characterizes the relative depths of the potentials. We suppose that the rotation occurs around the $\mathbf{r}=0$ point. By the definition of the rotated lattice it is periodic with periods $\mathbf{i}' = S\mathbf{i}$ and $\mathbf{j}' = S\mathbf{j}$:

$$V_2(\mathbf{r}) = V_2(\mathbf{r}+\mathbf{i}') = V_2(\mathbf{r}+\mathbf{j}'), \quad \text{and} \quad \mathbf{i}'\cdot\mathbf{j}' = 0. \tag{3}$$

It is convenient to associate continuous periodic potentials $V_1(\mathbf{r})$ and $V_2(\mathbf{r})$ with simplified discrete lattices, i.e. to represent the lattice in the form of atoms, whose positions coincide with maxima of $V_1(\mathbf{r})$,$V_2(\mathbf{r})$, with connections between nearest neighbours. To this end we define the lattice vectors

$$\mathbf{a}_{mn} = m\mathbf{i} + n\mathbf{j} \tag{4}$$

for the potential $V_1(\mathbf{r})$ and

$$\mathbf{b}_{m'n'} = m'\mathbf{i}' + n'\mathbf{j}' = m'S\mathbf{i} + n'S\mathbf{j} \tag{5}$$

for the potential $V_2(\mathbf{r})$. In (4) and (5) n, m, n' and m' are integers. The vectors $\mathbf{a}_{mn}$ and $\mathbf{b}_{m'n'}$ define discrete lattices associated with our periodic continuous potentials.

We now prove that: *the composite 2D potential* $V(\mathbf{r}) = V_1(\mathbf{r}) + V_2(\mathbf{r})$ *is periodic if and only if* $\cos\theta = a/c$ *and* $\sin\theta = b/c$, *where* (a,b,c) *is a Pythagorean triple.* Indeed, starting with the necessary condition we assume that $V(\mathbf{r})$ is periodic. Taking into account that by definition $\mathbf{a}_{00} = \mathbf{b}_{00}$, this assumption means that there exists at least one more site in each of the basic lattices such that the condition $\mathbf{a}_{mn} = \mathbf{b}_{m'n'} \neq 0$ is satisfied (i.e. sites of two basic lattices coincide in at least one more point - in schematic illustration presented in Fig. 1 such points are shown in black). The condition $\mathbf{a}_{mn} = \mathbf{b}_{m'n'}$ can be rewritten in the form

$$m = m'\cos\theta - n'\sin\theta, \quad n = n'\cos\theta + m'\sin\theta. \tag{6}$$

implying that $m^2 + n^2 = m'^2 + n'^2$. The system (6) can be considered as an algebraic system with respect to $\cos\theta$ and $\sin\theta$. Its solution exists when $n^2 \neq m^2$ and it can be written as

$$\cos\theta = \frac{mm' + nn'}{m'^2 + n'^2}, \quad \sin\theta = \frac{nm' - mn'}{m'^2 + n'^2}. \tag{7}$$

Let us define

$$a = mm' + nn', \quad b = nm' - mn', \quad c = m'^2 + n'^2. \tag{8}$$

Taking into account that $m^2+n^2=m'^2+n'^2$, it directly follows from (7) that

$$a^2+b^2=(m^2+n^2)(m'^2+n'^2)=c^2. \quad (9)$$

Therefore (a,b,c) is a Pythagorean triple, and the necessary condition is proven.

Now we turn to the sufficient condition. To this end, we have to set the rotation angle so that $\cos\theta=N_1/N$ and $\sin\theta=N_2/N$, where the integers $N_{1,2}$ and N satisfy the equation $N_1^2+N_2^2=N^2$, and prove that the superposition of two basic lattices set by vectors $\mathbf{a}_{nm}$ and $\mathbf{b}_{n'm'}$ is periodic. Since basic lattices are invariant with respect to translations by $m\mathbf{i}+n\mathbf{j}$ and $m'\mathbf{i}'+n'\mathbf{j}'$, it is enough to prove that there exist two equal noncollinear lattice vectors of the composite lattice. In other words, one has to prove that one can find two pairs of integers $(m_{1,2},n_{1,2})$ and $(m'_{1,2},n'_{1,2})$, so that the vectors $\mathbf{e}_1$ and $\mathbf{e}_2$ defined by

$$\mathbf{e}_1=\mathbf{a}_{m_1n_1}=\mathbf{b}_{m'_1n'_1}, \quad \mathbf{e}_2=\mathbf{a}_{m_2n_2}=\mathbf{b}_{m'_2n'_2} \quad (10)$$

are noncollinear. If this is proven, then $V(\mathbf{r})=V(\mathbf{r}+\mathbf{e}_1)=V(\mathbf{r}+\mathbf{e}_2)$, and hence $V(\mathbf{r})$ is a periodic potential.

Taking into account that now

$$S=\frac{1}{N}\begin{pmatrix} N_1 & -N_2 \\ N_2 & N_1\end{pmatrix},$$

the equality $\mathbf{a}_{mn}=\mathbf{b}_{m'n'}$ is guaranteed whenever the algebraic system

$$m=\frac{1}{N}(N_1m'-N_2n'), \quad n=\frac{1}{N}(N_2m'+N_1n') \quad (11)$$

makes it possible to find solutions in the form of a pair of integers (m,n) when another pair of integers (m',n') is fixed (or vice versa). For instance one finds one pair $(m,n)=(N_1,N_2)$ for $(m',n')=(N,0)$ and another pair $(m,n)=(0,N)$ for $(m',n')=(N_2,N_1)$. This choice of pairs of solutions is obviously not unique. The corresponding pair of lattice vectors $\mathbf{e}_1$ and $\mathbf{e}_2$ for composite potential $V(\mathbf{r})$ is given by

$$\mathbf{e}_1=N\mathbf{i}, \quad \mathbf{e}_2=N_2\mathbf{i}+N_1\mathbf{j}=S\mathbf{j}. \quad (12)$$

It is worth mentioning here that the vectors $\mathbf{e}_{1,2}$ are not just auxiliary objects that help to prove that a Pythagorean lattice is truly periodic. They are also a convenient tool for defining the primitive lattice cell. In fact they represent hypotenuses of two Pythagorean triangula with mutually orthogonal catheti (see e.g. the example below and Fig. **??**). Obviously the length of these vectors is defined by the third number in the corresponding Pythagorean triple (a,b,c), i.e. $|\mathbf{e}_{1,2}|=cd$, where d is the period of each of the sublattices.

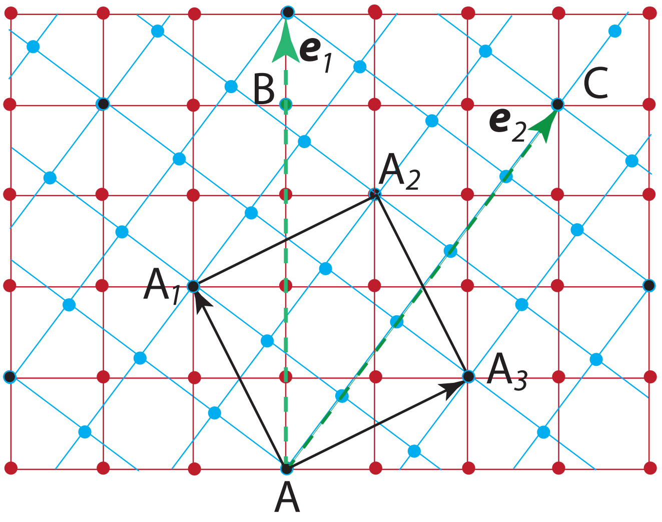

FIG. 1. (Color online) Pythagorean lattice obtained by the rotation of two discrete square lattices by the angle $\theta=\arctan(3/4)$ around the point A. Green vectors $\mathbf{e}_{1,2}$ are the ones used in the text for obtaining lattice periodicity. Black vectors $\vec{AA_1}$ and $\vec{AA_3}$ indicate the primitive cell. The "atoms" of two lattices are considered different and with black dots we indicate the locations where the sites of both sublattices coincide. The amplitude of a continuous Pythagorean lattice acquires maximal values around the black dots.

II. AN EXAMPLE

From the above analysis it follows that $\mathbf{e}_1$ and $\mathbf{e}_2$ can be viewed as the lattice vectors of a Pythagorean lattice. To make the correspondence between a continuous lattice and its discrete representation more obvious we indicate by blue and red dots maxima of both periodic sublattices. Whenever they coincide we obtain a site of the combined lattice; it corresponds to the local maximum of the composite continuous Pythagorean lattice. Generally speaking, $\mathbf{e}_1$ and $\mathbf{e}_2$ are not the vectors of a primitive cell of the lattice. As the case example lets us consider the discrete Pythagorean lattice generated by the triple $(3,4,5)$ as shown in Fig. 1, where two identical square lattices are rotated with respect to each other by an angle $\theta=\arctan(3/4)=\arccos(\mathbf{e}_1\cdot\mathbf{e}_2)$ (i.e. θ is the angle between vectors $\mathbf{e}_1$ and $\mathbf{e}_2$). The Pythagorean triangle generating this lattice is $\triangle ABC$. The knots or "atoms" of two sublattices forming a composite structure always coincide in the points characterized by the vector $m\mathbf{e}_1+n\mathbf{e}_2$, where m and n are integers. It turns out, however, that other points also exist, like A_1, A_2 and A_3 marked on the figure, where the atoms of two sublattices coincide. The existence of such points is ensured by the relation

$$2\varphi=2\arctan\left(\frac{1}{2}\right)=\arctan\left(\frac{4}{3}\right)=\frac{\pi}{2}-\theta$$

where φ is the angle between the vectors AA_1 and $\mathbf{e}_1$. Thus, the square $AA_1A_2A_3$ in Fig. 1 shows a square cell of the Pythagoren lattice, which is primitive, as is proven in the next section. Such a cell contains 9 "atoms" and its area always exceeds areas of primitive cells in the individual sublattices.

III. SQUARE CELL OF THE PYTHAGOREAN LATTICE

The example provided above illustrates a generic situation. More specifically, in this section we prove that:

(i) for an *arbitrary* Pythagorean lattice one can choose a *square* primitive lattice cell;

(ii) the area of such a square cell is not larger than the area of the cell defined by the lattice vectors (12);

(iii) the smallest among all congruent square cells is a primitive cell of the Pythagorean lattice.

In fact, the proof of this last statement also represents an algorithm of construction of the primitive cells for Pythagorean lattices.

To prove these statements, we should prove the existence of point(s) belonging to *both* square lattices, but at the same time deviating from the points defined by the vectors $\mathbf{e}_{1,2}$ given in (12), since cells defined by $\mathbf{e}_{1,2}$ are not square. Obviously, such points can be located only on the bisectors of the angles created by the axes of two sublattices. There are two such angles, one of which determines the Pythagorean triangle $\triangle ABC$ generating the lattice, as this is illustrated in Fig. 2. We denote the angle between the vectors $\mathbf{e}_1$ and $\mathbf{e}_2$ as 2φ. We also define the vector $\mathbf{b}_1$ along the angle bisector, i.e. the vector that forms angle $\varphi = \theta/2$ with each of the vectors $\mathbf{e}_{1,2}$. The projections of $\mathbf{b}_1$ on $\mathbf{e}_{1,2}$ have the length a. By definition we have $m^2 + n^2 = N^2$ and the following relation

$$\frac{n}{m} = \tan(2\varphi) = \frac{\tan\varphi}{1-\tan^2\varphi} \tag{13}$$

from which it readily obtained that

$$\tan\varphi = \frac{N-m}{n}. \tag{14}$$

Now our goal is to prove that a point O exists. It is defined by the vector $\mathbf{b}_1$ so that $|AD| = |AF|$ and $|OD| = |OF|$ are both integers, since in this case O will be the "atom" belonging to both sub-lattices. This is a trivial task in view of (14), because it is enough to choose $|OD| = |OF| = N - m < n$ and $|AD| = |AF| = n$. This proves statement (i).

Without loss of generality we further assume that $m > n$. Since each of the sublattices is square, there exists another atom O' belonging to two sublattices simultaneously and determined by the vector $\mathbf{b}_2$ (as shown in Fig. 2) that is orthogonal to $\mathbf{b}_1$ and has the same modulus, i.e. $\mathbf{b}_1 \cdot \mathbf{b}_2 = 0$ and $|\mathbf{b}_1| = |\mathbf{b}_2|$.

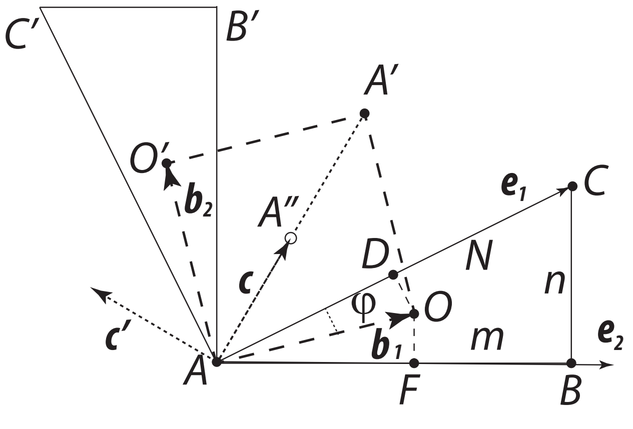

FIG. 2. (Color online) Two Pythagorean triangles orthogonal to each other are shown schematically. The orthogonal vectors $\mathbf{b}_1$ and $\mathbf{b}_2$ are possible choices for the primitive cell. For other notations see the text.

Next we verify that the area of the cell determined by $(\mathbf{e}_1, \mathbf{e}_2)$ is

$$V_e = N^2 \sin(2\varphi) = Nn$$

while the area of the cell determined by $(\mathbf{b}_1, \mathbf{b}_2)$ is

$$V_b = 2N(N-m) = 2N\left(N - m - \frac{n}{2}\right) + V_e < V_e$$

where we have used the inequality $N < m + n/2$ following from the estimate

$$\left(m + \frac{n}{2}\right)^2 = N^2 + n\left(m - \frac{3}{4}n\right) > N^2 + n(m-n) > N^2$$

(recall that we have chosen $m > n$). This proves statement (ii).

Next, using *reductio ad absurdum*, one can easily establish that the newly defined triangle $\triangle ADO$ (or, equivalently, $\triangle AFO$) is not Pythagorean. In fact, let us assume the opposite, i.e. that $\triangle ADO$ is Pythagorean. Then from the properties of the Pythagorean triples it follows that N should be odd. Thus if m is even, then $N - m = |OD|$ is odd and from $\triangle ADO$ we conclude that n is even. Similarly a supposition that m is odd leads to the conclusion that n is odd. However, it is known from the properties of Pythagorean triples (recall that $\triangle ABC$ is Pythagorean) that n and m cannot become even or odd simultaneously. Thus we have arrived at *absurdum.* This immediately leads to the conclusion that $|\mathbf{b}_{1,2}|$ is an irrational number (measured in the units of the square lattice constant).

If $N - m$ and n have a common largest integer divisor $p \in \mathbb{N}$, i.e. $N - m = pq_1$ and $n = pq_2$, with $q_{1,2} \in \mathbb{N}$ we introduce the vectors $\mathbf{b}'_{1,2} = \mathbf{b}_{1,2}/p$, which define a square cell congruent to the one based on $\mathbf{b}_{1,2}$. This newly defined cell has the smallest possible area among all congruent cells. Thus to conclude the proof, we have

to show that on the second bisector (passing through the point A and A' in Fig. 2) there are no points that simultaneously belong to both lattices and separated from A by a distance smaller than $|\mathbf{b}'_1| = |\mathbf{b}_1|/p$. We prove this for $p = 1$ (this makes it possible to refer directly to Fig. 2, while extension to $p > 1$ is straightforward and achieved by scaling p out).

Indeed, now $|\mathbf{b}_1|$ is the smallest distance between the neighbor lattice sites along the lines defined by $\mathbf{b}_{1,2}$. Once again, we use *reductio ad absurdum* and assume that on the line AA' (here we refer to Fig. 2), there is a point (say A'' shown in Fig. 2 by an empty circle) so that $c = |AA''| < |\mathbf{b}_1|$, where we use $\mathbf{c} = \vec{AA''}$. Since the 4-fold symmetry implies the existence of $\mathbf{c}' \perp \mathbf{c}$ with $c = c'$, the vector $\mathbf{b} = \mathbf{c} + \mathbf{c}'$ belongs to a line along either $\mathbf{b}_1$ or $\mathbf{b}_2$ and $|\mathbf{b}| = \sqrt{2}c$ (in Fig. 2 it is the line along $\mathbf{b}_2$). Since $|\mathbf{b}|$ cannot be smaller than $|\mathbf{b}_1|$ we know that c must belong to the interval $|\mathbf{b}_1|/\sqrt{2} < c < |\mathbf{b}_1|$. On the other hand, since A' is the lattice site, there must be verified $p_1 c = |AA'| = \sqrt{2}|\mathbf{b}_1|$ where $p_1 \in \mathbb{N}$ and $p_1 \geq 2$. Therefore, we conclude that $c \leq |\mathbf{b}_1|/\sqrt{2}$, which contradicts the previous conclusion. Thus, we have proven statement (iii).

IV. BANDSTRUCTURES OF THE PYTHAGOREAN LATTICES

The flatness of the first band is a generic feature of any Pythagorean lattices. In addition to the bandstructures of lattice corresponding to triple (3,4,5) as shown in the Fig. 5(a) of the main text, following we also show results corresponding to two other triples: (5,12,13) and (8,15,17).

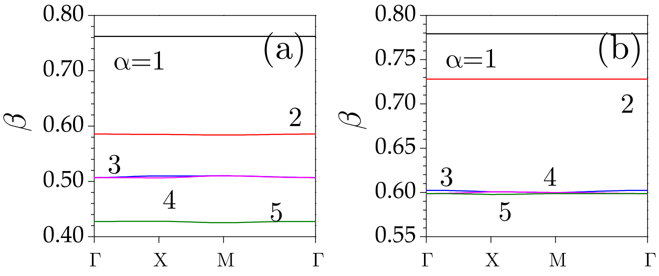

FIG. 3. (Color online) Band-gap spectrum of a periodic lattice with $\tan\theta = 5/12$ (a) and $\tan\theta = 8/15$ (b).

V. HEXAGONAL LATTICE

The most intriguing property of the Pythagorean lattice consisting in restoration of its periodicity for specific rotation angles of two sublattices is a general property that holds for sublattices of various symmetries, provided that they are periodic. However for sublattices different

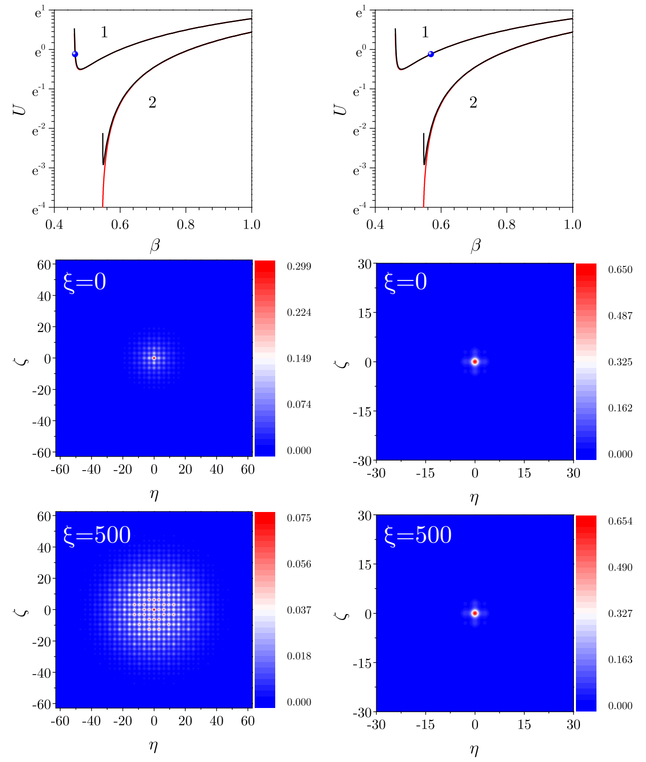

FIG. 4. (Color online) Evolution of two solitons in a composite potential in the presence of a self-focusing Kerr nonlinearity. The solitons have equal input powers ($U = 0.89$) but belong to the parts of $U(\beta)$ curve with different slope (first arrow, $\beta = 0.46$ (left), $\beta = 0.57$ (right)). The input and output pattern after a propagation distance of $\xi = 500$ are shown. $\theta = \pi/6$, $p_1 = 1$, $p_2 = 0.05$.

from square ones these specific angles are defined already not by Pythagorean triples, but rather by sets of three positive integers defined according to some rules that are unique for each particular type of sublattice. Here we describe such rules for hexagonal (or triangular) lattices, whose schematic sketch is presented in Fig. 5.

Now vectors of the primitive cell of each sublattice are given by $\mathbf{b}_{1,2}$ and $\mathbf{b}'_{1,2}$ (in Fig. 5 we show only one of the sublattices) and by α we denote the angle between them: for the hexagonal lattice $\alpha = \pi/3$. As before, we consider superposition of a given hexagonal lattice with another hexagonal lattice rotated by an angle θ with respect to the point A. Using the same arguments as in section I, to ensure periodicity of the composite structure we have to find two noncollinear vectors $\mathbf{e}_{1,2}$ so that [cf. (10), and see the example in Fig. 5]

$$\mathbf{e}_1 = m\mathbf{b}_1 + n\mathbf{b}_2 = m'\mathbf{b}'_1 + n'\mathbf{b}'_2, \quad (15)$$

$$\mathbf{e}_2 = m_1\mathbf{b}_1 + n_1\mathbf{b}_2 = m'_1\mathbf{b}'_1 + n'_2\mathbf{b}'_2. \quad (16)$$

where m, m', m'_1, m'_1, n, n', n'_1, n'_1 are integers. Similarly to the case of Pythagorean lattice, for $\mathbf{e}_1$ one can choose $m' = N$ and $n' = 0$. Then m, n, and N must satisfy the

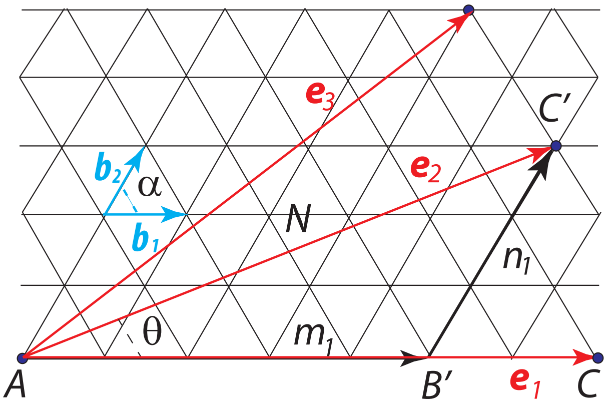

FIG. 5. (Color online) One of two triangular lattices with primitive lattice vectors $\mathbf{b}_1$ and $\mathbf{b}_2$ (shown in blue) and the angle α between them. Red vectors $\mathbf{e}_{1,2}$ are the lattice vectors defining a new composite periodic structure whose "atoms" are shown by the blue circles (to avoid overloading the figure with graphical details we show only the first nonrotated sublattice here). This example corresponds to the $(3,5,7)$ triple. We also show the vector $\mathbf{e}_3 = n\mathbf{b}_1 + m\mathbf{b}_2$ obtained upon exchange $m \leftrightarrow n$ in the expression for $\mathbf{e}_2$, i.e. the vector defined by the triple $(5,3,7)$. See text for the rest of notations.

equation

$$N^2 = m^2 + n^2 + mn. \qquad (17)$$

Furthermore, if θ is the rotation angle, by using the expression (16) for vector $\mathbf{e}_2$ and simple geometric considerations for triangle $\triangle AB'C'$ from Fig. 5, one can derive the following set of equations :

$$N\cos\theta = n_1 \sin\alpha, \quad N\cos\theta = m_1 + n_1\cos\alpha. \qquad (18)$$

It follows from (18) that

$$\sin\theta = \frac{n_1\sqrt{3}}{2\sqrt{n_1^2+m_1^2}}, \qquad \cos\theta = \frac{2m_1+n_1}{2\sqrt{n_1^2+m_1^2}} \qquad (19)$$

where m_1, n_1, and N must satisfy the equation

$$N^2 = m_1^2 + n_1^2 + m_1 n_1. \qquad (20)$$

From these arguments we conclude that the rotation angles leading to restoration of the periodicity in the superposition of two hexagonal lattices are fully determined by the triples of integers (m,n,N) satisfying the condition (17). These triples are obviously different from Pythagorean ones. In the Table V we show the lowest triples (ordered by the magnitude of m) and the corresponding rotation angles. Interestingly, the 8th and 9th lines of Table I show that in the case of hexagonal sublattices two different triples can correspond to identical rotation angles, the situation that can not be met in square Pythagorean lattices.

triple	$\sin\theta$	$\cos\theta$
$(3,5,7)$	$5\sqrt{3}/14$	$11/14$
$(5,16,19)$	$8\sqrt{3}/19$	$13/19$
$(7,8,13)$	$4\sqrt{3}/13$	$11/13$
$(7,33,37)$	$33\sqrt{3}/74$	$47/74$
$(9,56,61)$	$28\sqrt{3}/61$	$37/61$
$(11,24,31)$	$12\sqrt{3}/31$	$23/31$
$(11,85,91)$	$85\sqrt{3}/182$	$107/182$
$(13,35,43)$	$35\sqrt{3}/86$	$61/86$
$(14,16,43)$	$35\sqrt{3}/86$	$61/86$
$(14,66,74)$	$33\sqrt{3}/74$	$47/74$

TABLE I. Triples and corresponding rotation angles at which superposition of two hexagonal lattices becomes a periodic lattice.

VI. LDT IN TWO ROTATED HEXAGONAL LATTICES

As indicated in the previous section a composite lattice created by two mutually rotated hexagonal patterns becomes periodic for a strictly defined set of rotation angles (see Table I). To show how rotation angle and depth of second lattice affect localization of corresponding linear modes, let us consider a composite structure composed of two hexagonal lattices with the following specific realization, namely, $V(\mathbf{r}) = V_1(\mathbf{r}) + V_2(\mathbf{r})$, where

$$V_1(\mathbf{r}) = -p_1 \cdot \sum_{i=1,2,3} \cos[\Omega\cdot(\eta\cos\theta_i + \zeta\sin\theta_i)], \quad (21)$$

$$V_2(\mathbf{r}) = -p_2 V_1(S\mathbf{r}), \qquad (22)$$

here, $\theta_i = 0, 2\pi/3, 4\pi/3$ and $S = \begin{pmatrix} \cos\theta & -\sin\theta \\ \sin\theta & \cos\theta \end{pmatrix}$.

Now we calculate the form-factor for different θ and p_2 values using the same approach as for two square lattices. The dependence of the form-factor χ of the linear mode with highest eigenvalue on (θ, p_2) is shown in

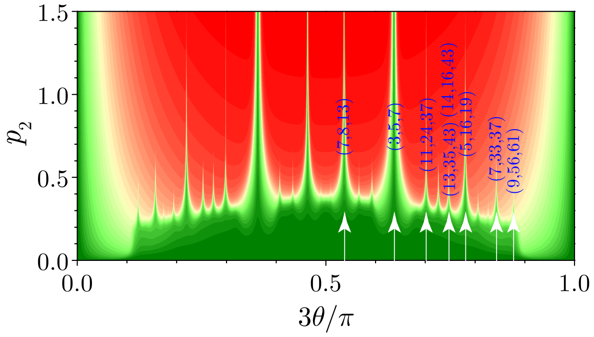

FIG. 6. $\chi(\theta, p_2)$ dependence illustrating LDT in the lattice created by two rotated hexagonal structures. White arrows indicate some of the angles at which periodicity restoration occurs. Triples associated with these angles are indicated too.

Fig. 4. Naturally, due to symmetry of the hexagonal lattice this dependence (Fig. 4) is now $\pi/3$-periodic. It clearly illustrates delocalization for a specific set of rotation angles, at which the lattice becomes fully periodic. Moreover, Fig. 4 also illustrates LDT upon gradual increase of the depth p_2 of the second lattice. Thus all effects observed for square patterns can be observed with hexagonal lattices, indicating the generality of the observed phenomenon.

VII. DYNAMICAL STABILITY OF SOLITON IN COMPOSITE POTENTIAL

In the main text we mentioned that the soliton stability in a medium with the focusing nonlinearity coincides with the so-called Vakhitov-Kolokolov stability criterion, namely, solitons are stable for the intervals where $dU/d\beta > 0$, and unstable where $dU/d\beta < 0$. Here we present two examples of soliton evolution in the potential with a self-focusing nonlinearity. The shown solitons have the same power value but locate at different points of the $U(\beta)$ branch (see the first arrow of Fig. 4). From the evolution shown we observe that soliton on the part of $U(\beta)$ with negative slope quickly loses its initial shape and expands significantly. In contrast, a soliton whose propagation constant belongs to the positive slope of $U(\beta)$ maintains its shape over a long propagation distance (the whole distance computed numerically).

ACKNOWLEDGMENTS

The work of C. Huang and F. Ye has been supported by the NSFC, Grants Nos. 11104181 and 61475101. V. Konotop acknowledges support of the FCT (Portugal) grants UID/FIS/00618/2013. The work of Y. Kartashov and L. Torner has been partially supported by the Severo Ochoa Excellence program.
